# Supplementary material for: A non-canonical role for UPRER during heat stress in C. elegans
Source: bioRxiv. 2026 Jan 9:2026.01.08.698479. Preprint. [Version 1] doi: 10.64898/2026.01.08.698479 (PMC12803219; doi:10.64898/2026.01.08.698479)
Supplement: 1 [file NIHPP2026.01.08.698479v1-supplement-1.pdf]

bioRxiv preprint doi: https://doi.org/10.64898/2026.01.08.698479; this version posted January 9, 2026. The copyright holder for this preprint (which was not certified by peer review) is the author/funder, who has granted bioRxiv a license to display the preprint in perpetuity. It is made available under aCC-BY 4.0 International license.

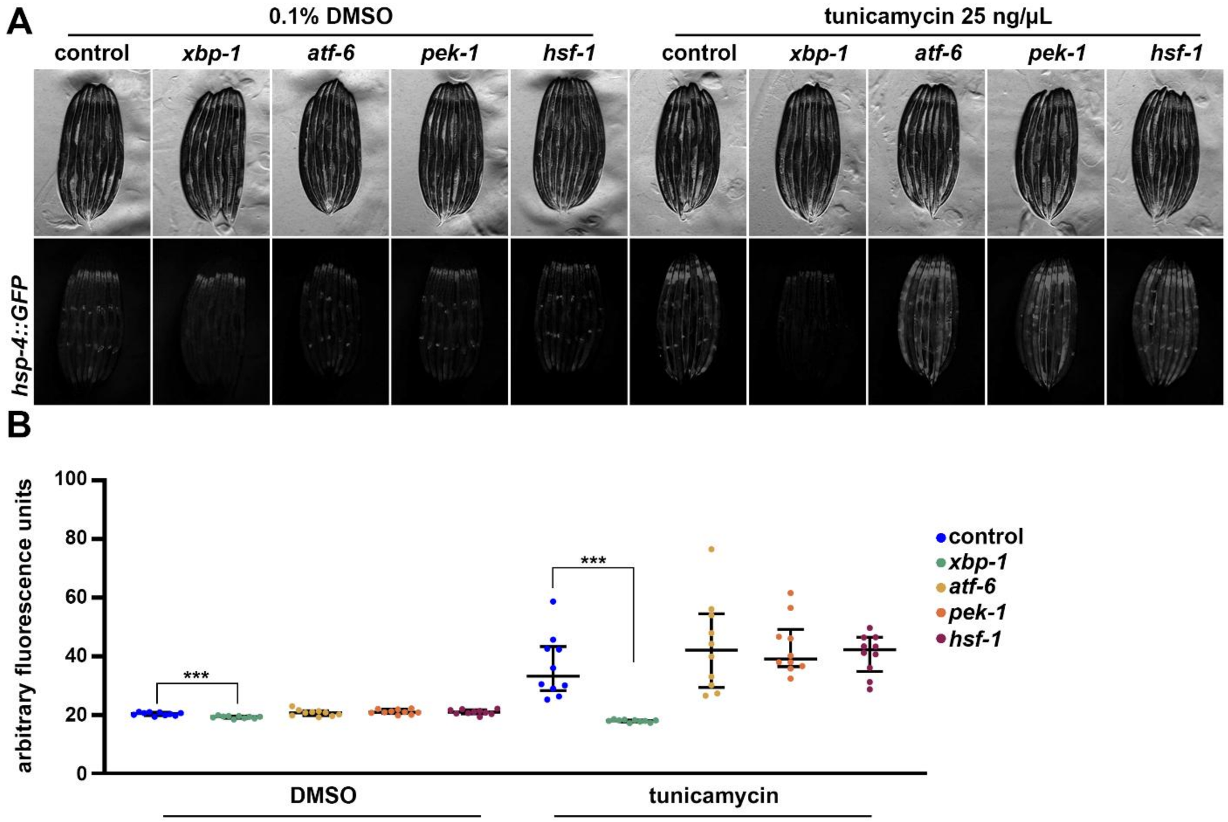

Fig. S1. *hsf-1* is not required for ER stress induced UPR^{ER}. (A) Representative fluorescent images of day 1 adult animals expressing *hsp-4p::GFP* grown on EV (control), *xbp-1, atf-6, pek-1,* or *hsf-1* RNAi bacteria from L1. L4 animals are treated with 0.1% DMSO or 25 ng/µL tunicamycin in M9 solution in a rotator at 20 °C for 4 hours. DMSO or tunicamycin are washed 3x with M9 solution and animals are placed on standard RNAi plates with EV RNAi bacteria and grown at 20 °C to recover overnight (maximum of 16 hours). Animals are imaged on day 1 of adulthood. Data is representative of 3 independent replicates. **(B)** Quantification of A represented as arbitrary fluorescent units, which are integrated fluorescence intensity measurements using Image J. Data is representative of three biological replicates where individual dots represent individual animals and lines represent median plus interquartile range. *** = $p < 0.001$ using Kruskal-Wallis multiple comparison testing.

bioRxiv preprint doi: https://doi.org/10.64898/2026.01.08.698479; this version posted January 9, 2026. The copyright holder for this preprint (which was not certified by peer review) is the author/funder, who has granted bioRxiv a license to display the preprint in perpetuity. It is made available under aCC-BY 4.0 International license.

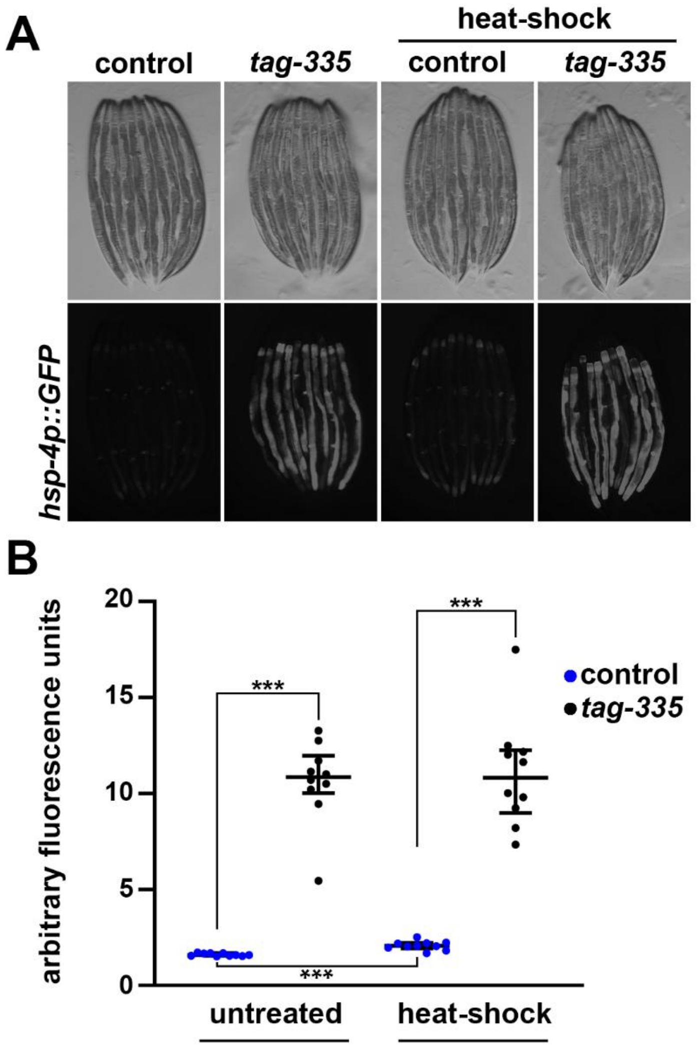

Fig. S2. ER and heat stress mediated UPR^{ER} are not additive. (A) Representative fluorescent images of day 1 adult animals expressing *hsp-4p::GFP* grown on EV (control) or *tag-335* RNAi bacteria from L1. Heat-shock conditions are 2 hours at 34 °C followed by a 4-hour recovery at 20 °C. Animals are imaged on day 1 of adulthood. Data is representative of 3 independent replicates. **(B)** Quantification of A represented as arbitrary fluorescent units, which are integrated fluorescence intensity measurements using Image J. Data is representative of three biological replicates where individual dots represent individual animals and lines represent median plus interquartile range. *** = $p < 0.001$ using Kruskal-Wallis multiple comparison testing.

bioRxiv preprint doi: https://doi.org/10.64898/2026.01.08.698479; this version posted January 9, 2026. The copyright holder for this preprint (which was not certified by peer review) is the author/funder, who has granted bioRxiv a license to display the preprint in perpetuity. It is made available under aCC-BY 4.0 International license.

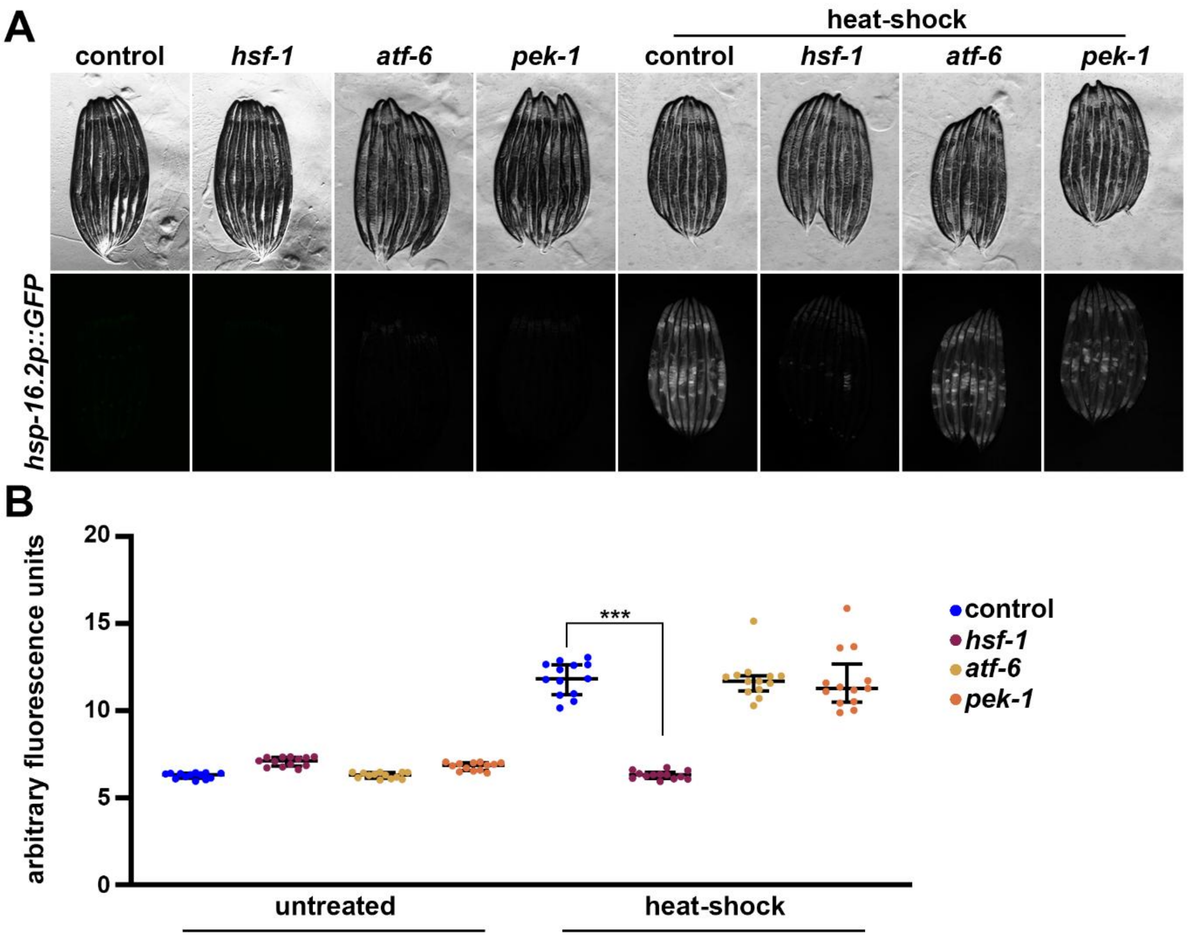

Fig. S3. The HSR requires *pek-1* and *atf-6*. (A) Representative fluorescent images of day 1 adult animals expressing *hsp-16.2p::GFP* grown on EV (control), *hsf-1, atf-6,* or *pek-1* RNAi bacteria from L1. Heat-shock conditions are 2 hours at 34 °C followed by a 2-hour recovery at 20 °C. Animals are imaged on day 1 of adulthood. Data is representative of 3 independent replicates. **(B)** Quantification of A represented as arbitrary fluorescent units, which are integrated fluorescence intensity measurements using Image J. Data is representative of three biological replicates where individual dots represent individual animals and lines represent median plus interquartile range. * = $p < 0.05$; *** = $p < 0.001$ using Kruskal-Wallis multiple comparison testing.

bioRxiv preprint doi: https://doi.org/10.64898/2026.01.08.698479; this version posted January 9, 2026. The copyright holder for this preprint (which was not certified by peer review) is the author/funder, who has granted bioRxiv a license to display the preprint in perpetuity. It is made available under aCC-BY 4.0 International license.

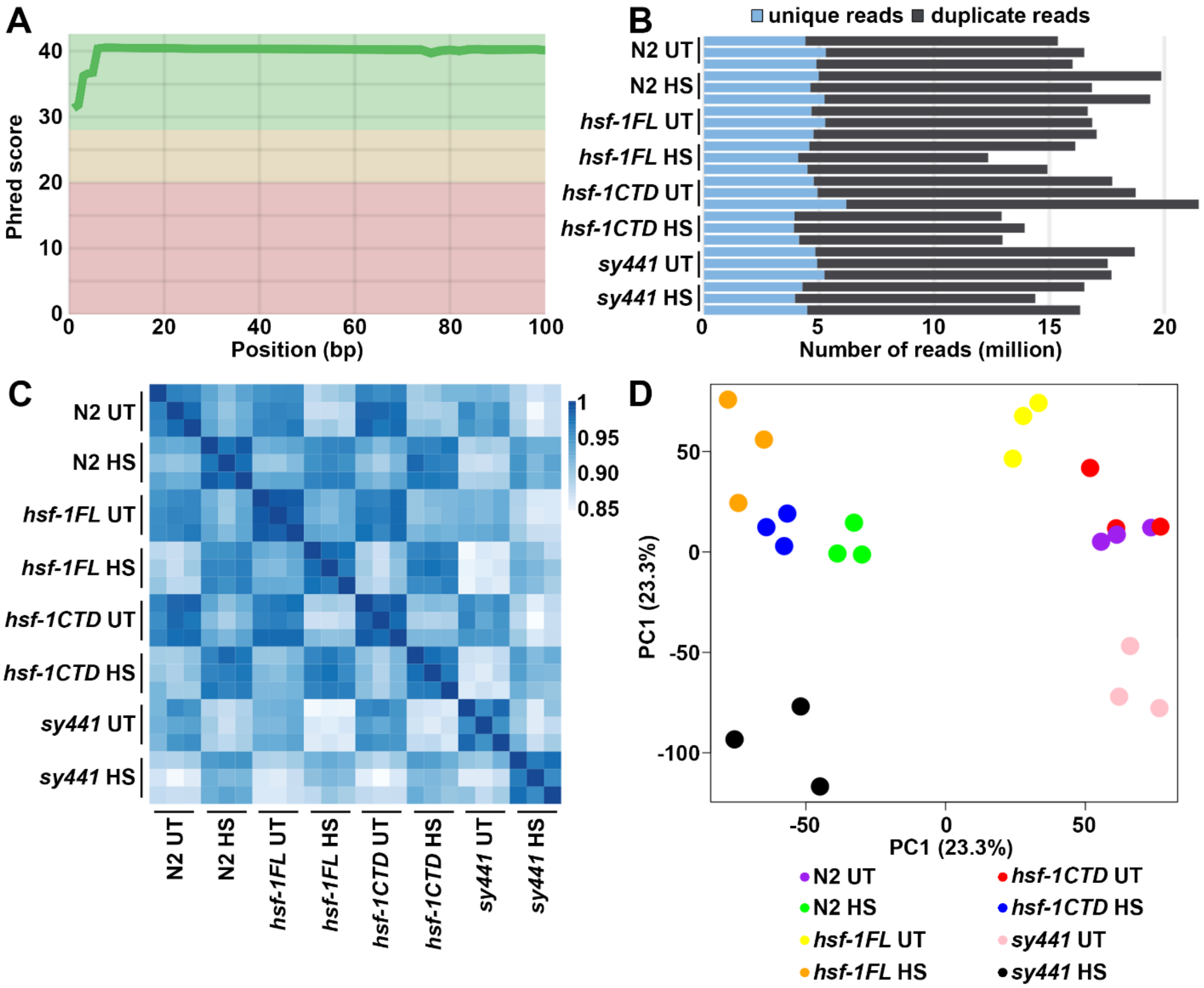

Fig. S4. Quality control for RNA-seq libraries of heat-shock activation in *C. elegans*. (A) Mean quality score (Phred score) of each RNA sequencing library. X-axis indicates the base pair (bp) position of each sequence and the y-axis indicates the Phred score. The graph was generated by MultiQC(Ewels et al. 2016). **(B)** The number of unique (blue) and duplicated (black) reads from each pair-wise sequencing library (n = 3). **(C)** Spearman correlation plot of all RNA sequencing libraries (n = 3). **(D)** PCA plots of all RNA sequencing libraries.

bioRxiv preprint doi: https://doi.org/10.64898/2026.01.08.698479; this version posted January 9, 2026. The copyright holder for this preprint (which was not certified by peer review) is the author/funder, who has granted bioRxiv a license to display the preprint in perpetuity. It is made available under aCC-BY 4.0 International license.

Table S1. Table of all significant GO terms. All significant GO terms for for N2, *rab-3p::hsf-1FL*, *rab-3p::hsf-1CTD*, and *hsf-1(sy441)* animals for all significant DEGs of heat-shocked vs. untreated controls for each condition.

Table S2. DEGs of HS vs. UT. All differentially expressed genes represented as $\log_2$(fold-change) for N2, *rab-3p::hsf-1FL*, *rab-3p::hsf-1CTD*, and *hsf-1(sy441)* animals calculated as heat-shocked vs. untreated controls for each condition.

Table S3 DEGs for UPR heat map. All $\log_2$(fold-change) used to create heat-map of **Fig. 5A**.

Table S4. DEGs for XBP-1 targets heat map. All $\log_2$(fold-change) used to create heat-map of **Fig. 5B**.

Table S5. DEGs against N2 control. All differentially expressed genes represented as $\log_2$(fold-change) N2, *rab-3p::hsf-1FL*, *rab-3p::hsf-1CTD*, and *hsf-1(sy441)* animals with heat-shock (HS) or left untreated (UT) calculated against N2 controls.
